# Considerations for the Role and Implementation of Reproductive Health Shared Decision‐Making Interventions for Cystic Fibrosis: A Qualitative Investigation of Perspectives From Clinicians and Females With Cystic Fibrosis

**DOI:** 10.1002/ppul.71643

**Published:** 2026-05-05

**Authors:** Sarah Brown, Denitza Williams, Jamie Duckers, Traci M. Kazmerski, Olivia M. Stransky, Caitlin Mason, Alexis Bennett, Jennifer Ward, David Taylor‐Robinson, Oluwaseun B. Esan, Shantini Paranjothy, Daniela Schlüeter, Rhiannon Phillips

**Affiliations:** ^1^ School of Sport and Health Sciences, Cardiff Metropolitan University Cardiff UK; ^2^ Division of Population Medicine School of Medicine, Cardiff University Cardiff UK; ^3^ All Wales Adult CF Centre, Cardiff and Vale University Health Board Cardiff UK; ^4^ Department of Pediatrics University of Pittsburgh School of Medicine Pittsburgh USA; ^5^ Department of Public Health Policy and Systems, University of Liverpool Liverpool UK; ^6^ Aberdeen Centre for Health Data Science, Institute of Applied Health Sciences, University of Aberdeen Aberdeen UK

**Keywords:** cystic fibrosis, decision aids, implementation, qualitative, reproductive health, shared decision‐making

## Abstract

**Introduction:**

Advances in cystic fibrosis (CF) treatment have seen more people with CF starting a family. Females with CF (FwCF) have expressed a need for improved reproductive health‐related shared decision‐making (SDM) with health care providers. This study explored perspectives of FwCF and clinicians on the role and implementation of SDM tools in CF reproductive health care.

**Methods:**

We conducted qualitative semi‐structured interviews with FwCF and clinicians. We recruited clinicians through professional networks and FwCF via a previous study and social media. Interviews explored experiences of SDM related to reproductive health. Participants were given examples of SDM tools and asked to consider their utility within CF care. We analyzed interview transcripts using an inductive approach, identifying key themes.

**Results:**

Six FwCF and 23 clinicians participated. Four themes were identified: Perceived usefulness of an SDM approach for CF reproductive health, Role of tools in facilitating SDM, Considerations for SDM tool development, and Implementation of SDM in routine CF care. Participants saw information provision as key to SDM, enhancing patient confidence to initiate and engage in conversations. Participants considered SDM tools helpful at three stages: preparing for consultations, facilitating in‐consultation communication, and supporting post‐consultation reflection and discussion. Trustworthy content was key to engagement. Participants felt reproductive health conversations should start in adolescence and recur throughout the life course. Clinicians highlighted the need for a service‐wide culture supporting SDM.

**Conclusions:**

Both groups supported early and ongoing reproductive health‐related SDM. Further research should evaluate CF‐specific reproductive health SDM interventions that build confidence, skills, and foster a health‐service culture supportive of SDM.

## 1 Introduction

The introduction of cystic fibrosis transmembrane conductance regulator protein (CFTR) modulators has improved health and life expectancy for people with cystic fibrosis (CF) [1, 2]. Consequently, more females with CF are considering starting a family [3, 4]. CF is associated with reproductive and parenting challenges influenced by maternal lung function, the effects of medications on pregnancy and breastfeeding, and the health impact of caring for a baby [5, 6]. Individuals with CF with the potential to become pregnant need to be equipped to make informed choices regarding their reproductive health [7, 8]. Reproductive health decisions are complex and sensitive for this population, and females with CF have expressed a need for further support through shared decision‐making (SDM) [9, 10]. While CF management provides multiple opportunities for SDM, research suggests that people with CF and clinicians do not always participate equally in decision‐making [10, 11, 12].

Decision aids are tools that aim to help people make informed health‐related choices. They can support SDM and have been shown to increase positive outcomes for both patients and clinicians [13]. However, there is a need for further exploration of their use within reproductive health and long‐term care [14]. This includes consideration of where such tools optimally sit within the complex and longitudinal SDM process [15, 16]. In this study, we explored the perspectives of FwCF and clinicians on the role and implementation of SDM tools related to reproductive health in CF. The term “clinician” is used throughout to describe an individual with a patient‐facing role involved in the care of FwCF.

## 2 Materials and Methods

### 2.1 Design

We conducted this study in the United Kingdom (UK), where medical treatment is provided free at the point of care. In the UK, CF is usually managed by a multidisciplinary team in Secondary or Tertiary (i.e., highly specialist) care settings, although elements of reproductive health (such as contraception) may be managed in Community or Primary Care settings.

We used a qualitative research design, conducting online one‐to‐one semi‐structured interviews for both FwCF and clinicians. We used topic guides and prompts to explore participants' experiences and views on the implementation of SDM in routine health care and the role of SDM tools. We provided three tools in advance of the interview and used them as prompts to facilitate discussion: *MyVoice:CF*, an online interactive tool developed in the United States for FwCF, providing information on multiple aspects of reproductive health from contraception to pregnancy and postpartum [17], a reproductive health downloadable booklet from a UK‐based charity (the CF Trust) [18] and a generic decision aid template (Ottawa Personal Decision Guide) [19]. These tools were chosen to provide a variety of lengths, modes of access, and content. As the *MyVoice: CF* tool was designed for FwCF, we focused our recruitment on females with pregnancy potential, to ensure that this resource was not being used outside its original scope. Ethical approval was granted by Cardiff Metropolitan University Ethics Committee (Ref PGR‐5903). All participants provided written consent.

### 2.2 Participants and Sampling

We recruited clinician participants by email via professional networks. FwCF were recruited using convenience and snowball sampling. We contacted females with CF who participated in a previous qualitative study exploring reproductive goals [20] and had expressed an interest in participating in further research. The study was also advertised via social media and online CF support groups (i.e., Facebook, X, Instagram). We determined sample sizes pragmatically using the concept of “information power” [21]; the specificity of the research topic and population suggested that the sample would provide sufficiently rich data to meet the study aim.

Any clinician who provided direct care for individuals with CF in a UK setting was eligible to participate. Inclusion criteria for FwCF were the potential to become pregnant, a diagnosis of CF, at least 18 years old, resident in the UK. Screening questions were used at the recruitment stage, asking potential participants to confirm they had been diagnosed with CF by a doctor and to capture information about CF‐specific prescribed medication; however, diagnosis of CF was not independently verified (e.g., through clinic records). FwCF were given a 10 GBP gift voucher to thank them for participating.

### 2.3 Data Collection

We conducted interviews online using Microsoft Teams, recorded and transcribed verbatim. No field notes were taken. JW and AB, female non‐clinician experienced qualitative postdoctoral researchers, conducted the clinician interviews; CM (female MSc Health Psychology student) interviewed FwCF. None of the researchers had met the interviewees before the interview. Interviewers introduced themselves to participants at the start of the interview and described their role in the research team.

### 2.4 Clinicians

We asked clinicians to describe their role and setting. They were given all three SDM tools prior to the interview, with access to the full interactive functionality of *MyVoice:CF* in “preview mode” (i.e., no data was collected while they explored the tool).

### 2.5 Females With CF

We gathered data on age, ethnicity, education, relationship status, number of children, and whether they wanted (more) children. Some clinicians had found it challenging to consider all three tools before the interview; therefore, to reduce participation burden, FwCF were only shown the *MyVoice:CF* decision support tool, as this was the tool discussed most in the clinician interviews. They were given access beforehand and asked to revisit the tool during the interview. Participants were free to view any aspect of *MyVoice:CF* and were not time‐restricted. They were encouraged to ‘think aloud’, sharing their thoughts. We chose this approach to maintain focus on the SDM tool while situating the comments in a wider discussion about SDM, providing a deeper understanding of the use of SDM tools in the context of reproductive health for FwCF [22].

### 2.6 Data Analysis

Initially, data were analyzed separately for each group, before being synthesized to determine commonalities and discrepancies in identified themes. We took an inductive thematic analysis approach initially. This involved familiarization with data; generation and definition of codes; and construction, review, and refinement of themes [23]. In synthesizing the datasets from clinicians and FwCF, two researchers generated a coding framework bringing together the themes from the initial analyzes. NVivo qualitative data management software was used throughout. The coding framework was further developed and refined through discussion. This approach to qualitative data synthesis has been used previously for developing complex health interventions [24].

## 3 Results

We interviewed six FwCF and 23 clinicians (See Tables 1 and 2 for demographics). Interviews lasted between 38 and 146 minutes. No participants withdrew from the study, and no repeat interviews were carried out.

**Table 1:** Participant demographics: people with cystic fibrosis.

Variable	Category	PwCF *N* = 6 *N* (%)
Ethnicity	White British	5 (83.3)
	White other	1 (16.7)
Relationship status	Single	1 (16.7)
	Engaged	2 (33.3)
	Married	3 (50)
Level of education	Level 3 (A‐level)	2 (33.3)
	Level 6 (undergraduate degree)	2 (33.3)
	Level 7 (postgraduate degree)	2 (33.3)
Current number of children	0	4 (66.7)
	1	1 (16.7)
	3	1 (16.7)
Want children now or in the future	Yes	2 (33.3)
	No	2 (33.3)
	Not sure	2 (33.3)
Age	Range: 23–42 years	Mean 32.7 (SD 6.6)

**Table 2:** Participant demographics: clinicians.

Role	Clinicians *N* = 23 *N* (%)
Consultant (the most senior grade of doctor, listed on the General Medical Council specialist register)	8 (34.7)
Doctor (working at a level of responsibility below a consultant)	3 (13.0)
Clinical Nurse Specialist	5 (21.7)
Physiotherapist	3 (13.0)
Pharmacist	1 (4.4)
Dietician	1 (4.4)
Social worker	1 (4.4)
Youth worker	1 (4.4)
*Years working in CF care*	
0–5	5 (21.7)
5–10	6 (26.1)
10+	8 (34.7)
Not provided	4 (17.4)

We identified four themes from the combined data: Perceived usefulness of an SDM approach for reproductive health, Role of SDM tools in facilitating SDM, Considerations for SDM tool development, and Implementation of SDM in routine CF care. See Table 3 for indicative quotes.

**Table 3:** Qualitative analysis themes and indicative quotes.

Main theme	Sub‐theme		*Patient quotes*	*CF health care team quotes*
The perceived usefulness of an SDM approach for sexual and reproductive health	Inherent to practice *This sub‐theme describes the conflicting experiences of SDM within usual practice*.		*…you see your doctor every four weeks, like you are already… dependent on, dependent on which CF team it is…and the patient. But you're pretty used to advocating for yourself and being a, very much a part of your own care and every decision is, We think this, what do you think? (P3, 31, 3 children)* *No one's really ever sat down formally with me and said, like, okay, these are the things we would need to do. (*P5, 29, no children). *So, I haven't had, had an in depth like conversation about it [reproductive health]. But then I haven't had any like, because I'm not planning on having a family right now, I've not like had any, like, yeah, no, in depth conversations.* (P1, 23, no children)	*I think it happens every day, and you can't necessarily always plan for it, and sometimes it will just happen naturally…* (HP1004, Physiotherapist) *So shared decision‐making to me; I suppose it's two aspects. It's one, first of all, making sure that you take into account patients values, what their preferences are, if they have any strong opinions about certain treatments and also making sure that shared decision‐making also is within the MDT as well, cause there'll be certain factors that I may not be aware of and I'll need to liaise with them in terms of, like, their social background, their history as well. So shared decision‐making is making sure everyone's involved and making sure that values are respected, but also making sure we do what's best for the patient as well, making sure that's communicated to the patient as well. So we have a decision made all together from all avenues. It's not just one person leading, it has to be taken into account from various aspects.* (HP1024, Pharmacist)
	Supports the patient‐HCP relationship *This sub‐theme encapsulates the belief that SDM enhances and strengthens the patient‐HCP relationship through open, honest conversations*.		*my health was better at, at the time…But it, they knew it would deteriorate, so they said…pregnancy will be fine, but having a baby and having a child will really, really damage your health and you need to know that and appreciate that it is… you are really gonna cut your life expectancy down with a child. But, we'll support you if that's what you want and we'll support you the whole way through. (*P3, 31, 3 children*)*	*I guess it makes that patient feel that their kind of views are really important. And by making, by making them feel involved in the process, they're much more likely to actually adhere to any plans that are made, and to kind of, a better level perhaps, of respect between the clinician and the patient, because they'll both have heard kind of, each other's viewpoints on things and understand where perhaps, comments are coming from and discussions are coming from. They might choose to ignore them, but at least that's an active choice, rather than one coming from lack of understanding and knowledge.' (*HP1006, Physiotherapist*)* *for example, if we talked about an important topic like transplant for example, you know, we recognize that it's not a one‐off, like a 1‐h conversation with them and, and kind of that's it, you need to make a decision. I, I think that sometimes those discussions are a bit of a journey, so it's a bit of a, a bit of a marathon if you see what I mean, or a 10 K run I guess, rather than a sprint… it's important that we then work with the patient along the way until they make a decision.* (HP1011, Doctor)
	Providing information *This sub‐theme identifies information provision as a key aspect of the SDM process*.		*Yes, it's interesting because the only time I ever really talked about pregnancy with my CF clinic was when they were basically explaining like, these are the risks, and you know, this is what could happen. But now obviously, with Kaftrio, it's a little bit different…* (P5, 29, no children) *It's normally just, if you get pregnant, this is kind of the genetic risk, or you know, rather than actually, well, this is what it would look like, the whole journey…*. (P6, 37, no children)	*sometimes that will involve kind of providing further information to patients to help them make decisions. Generally, a lot of our patients feel that they're quite well‐educated in CF and CF care and their own health, but I am continually surprised, actually, about patients gaps in their knowledge or misunderstandings or information they just haven't retained. So yeah, we do a lot of education and repeating education about things to make sure that the decisions they're making, both in hospital and at home are based on factual knowledge, and not practice their opinion and experience.* (HP1006, physiotherapist)
The role of decision support tools in facilitating SDM	Preparing for a decision‐focussed conversation *This sub theme collates participant views that preparing for a conversation is a key role for SDM tools. This was further expanded into two distinct aspects of preparation: building a knowledge base and initiating a conversation*.		*I've just never, never even thought about it. I mean, I would hope that there wouldn't be enough chance that I would get pregnant by accident. But now I've thought about it. That would be ridiculously irresponsible if I did. Yeah, never thought about that*. (P4, 34, no children) *Yeah, I think it's easier to have it* [information] *all in one place. Because I don't know about other people's teams, but it is something in my team that all the team have very different opinions on*. (P4, 34, no children)	*I am not sure that they're gonna have a huge role in the consultation myself. There are lots of things to go through in a CF clinic appointment with the multi‐system nature of the condition. I think… I personally think it might be more helpful for the patient to be directed to it, to go through it at their own pace and looking at the things that they're asked… they're interested in or have concerns about in advance of the clinic*. (HP1014, consultant) *So I know this is a tool that is made for patients, I get that but I'm sure many of my colleagues will learn a lot from reading it. It's difficult to force people. I can't make it part of their mandatory training, but I think disseminating the information, having the tool spoken about and be readily available, will trigger, if you like, clinicians to look it up to read more about it.’* (HP1023, consultant) *you don't know what you don't know until someone gives you information* (HP1006, Physiotherapist)
	As an in‐consultation communication aid *This sub theme collates participant views that SDM tools play a role as a communication aid within a consultation. Two aspects of this were identified: the utility of SDM tools to guide a conversation and to tailor information to the individual*	Initiating a conversation *This aspect of preparation describes how SDM tools could increase confidence to initiate an SRH‐focussed conversation*.	*it would make me feel more confident because I would have more information up front. And I'm not just sat there asking questions, and I don't really know anything about or missing questions entirely not asking the things I should be saying. This will give me a much better like arsenal to go and then present my own worries or concerns or whatever, to my doctor, then yeah, we can work through it that way*. (P5, 29, no children)	*So things like posters in clinics, leaflets in clinics about pregnancy and decision‐making, that they can independently kind of, access it if they wish, so that we kind of empower them to feel confident in, in asking to have those conversations, if we've not really picked up on it ourselves*. (HP1006, physiotherapist)
		Guide a conversation *This aspect describes using an SDM tool as a communication aid within a consultation, providing structure, identifying topics of relevance and ensuring a comprehensive discussion*	*…talking about family planning can be a little bit, you know, hard to kind of reach that subject, or if you're not feeling, what kind of words to say, or how to ask these questions, at least this [SDM tool] can give you a bit more of like a private way of understanding and breaking down the questions that you have in your own head*. (P5, 29, no children) *Because, you know, I'm sure some people don't like talking about this. So being able to then just kind of discreetly hand them a list of things that you do want to know and then they can give you more information about it, I think is really good*. (P5, 29, no children)	*it [SDM tool] could be timesaving, because it's a good way of providing information, an effective way of providing information, and perhaps an easy way of guiding conversations with patients, and highlighting, kind of what they, what areas they may be lacking in information. I guess the, from the clinician's point of view, it's a better use of resources. Because if a patient, if a patient comes to a clinic prepared with, with the kind of thoughts already around pregnancy and what they want to discuss as well, then it's a better use of everyone's time*. (HP1006) *…so the advantage is it ensures that we are more thorough, that we don't miss important aspects of it (*HP1011, consultant*)* *Sometimes it [SDM tool] also takes the lid off things that perhaps patients aren't ready to discuss at that point in the conversation.* (HCP1007, Consultant).
		Tailoring information *This aspect describes using SDM tools to provide CF‐specific information and to tailor information to the individual*.	*I think I would have appreciated if they'd said about the contraception choices, because I still don't know about them now, it's not really like, well, I can't really go like Google it and be like, oh, like, what's my contraceptive choices for someone with CF? You know, like, it's not, I don't think it's there. (P1, 23, no children)* *I don't know about other people's teams, but it [pregnancy] is something in my team that all the team have very different opinions on* (P4, 34, no children)	*you can't always, you know, really account for every individual situation. So there will be people in these kind of things, who will be on a range of medications, etc. which may make things quite dangerous, but nothing can get as specific as that. So I think the one way to do it is to ensure that, you know, there's always a referral back to kind of, the health care team*. (HP1016, consultant) *I think, making sure that people have got access to information and having a bit more structure around the decision‐making is helpful. Because anecdotally, from what I hear from patients who are on various Facebook groups or other online forums, around pregnancy and fertility, there does seem to be variability in the sort of information that they've been given from CF teams…* (HP1014, Consultant) *when they've not got a person there they can specifically ask questions to, it [decision‐support tool] can kind of* —*it has the potential to, to let worries or concerns kind of become bigger, and then kind of scare them about the subject more than needs be, whereas if you're talking with someone face‐to‐face, you can be like, Oh, you just said this, can you explain it to me again? So there's kind of less room for confusion* (HP1009, F2 Doctor)
	Supporting reflection and deliberation *This sub‐theme discusses how an SDM tool could encourage reflection and deliberation pre‐ or post‐ consultation*.		*You might even find that when you go through it again, that something might have changed when you re‐read it. So you might have done it all, and then you read it all, and you think well actually, maybe I've changed my mind a little bit on a section*. (P4, 34, no children) *‘I think it's good to go through it by myself, then I can be like, okay, hey, you know, go to my partner and say, hey, let's look at this, like, this is some cool information. And then from that, the next thing would be like, I go through it with my mum or whatever, and then maybe the doctor after that, or even a friend, I think being able to show it to people is really useful because then people can, you know, they can also learn about it and get all that kind of information*.’(P5, 29, no children)	*people in that consultation, they're not gonna remember everything you say, and it can be quite overwhelming if it's a topic you haven't discussed before. And so giving people information that they can take away and read and come back to you later, make notes on at the time, is always gonna be a good thing* (HP1009, F2 Doctor). *it does allow the person with CF to take it away to have conversation, informed conversations with other key stakeholders in their life, be that partners, parents, key friends, who, who can't be there in the consultation, it allows them to share that information with those other key people’* (HP1015, Consultant) *‘I guess the assumption is that by using some of the tools with lots of information on it, you assume that the patient has read them. You assume that they have understood them as, in the way that they're intended to be understood…So I guess there is an importance to kind of check, actually, what has been read and what the interpretation has been, and to encourage conversations around it to check that.’* (HCP1006, Physiotherapist)
Considerations for support tool development	The importance of feeling represented *This sub‐theme considers the importance of SDM tool content relatability through culturally appropriate terminology and language, representations of ethnic diversity, gender or relationship diversity and patient stories*.		*So you could tell some of it is still American, like, the wording. And it wouldn't maybe apply to us in the UK*. (P6, 37, no children) *sometimes I think it might be nice to be, if there was something where almost a bit like other people's, like either people's stories, star… like sort of an honest outlook on it, not just a medical outlook on it.* (P4, 34, no children)	*I mean, this is an American tool, first of all. It's not directly applicable to our health care system*. (HP1013, consultant) *I particularly like it when they have individualized stories, because you can then, it becomes much more relatable, so I know, a bit further on in there, it's, you know, they have examples of the, the experiences of individual people with CF and I think that makes it much more personal and you know, you might be able to go away and actually contact that person as well, because a lot of them have a social media presence*. (HP1019, consultant)
	Trustworthy *This sub‐theme discusses the importance of SDM tool trustworthiness, through endorsement or development by a credible source and ensuring content is current*.		*The CF team need to be telling you about it [SDM tool]. Because if the CF team tells you something it gives it credibility, you know it's worth doing.’* (P2, 42, one child) *You kind of trust it more if it's from the UK, because it's like, current, it's like more tailored to you.* (P1, 23, no children)	*So at an organizational level, I think, you know, if there's something nationally that we can all just use, that's free to access given the fact that we work in the NHS, then I think everyone would jump at it. It's really, you know, if there's something centralized from a trusted source, like the CF Trust, that we can send patients too and say, look at this, read this, and let's make an appointment in two months' time…* (HP1019, Consultant)
	Format *This sub‐theme encapsulates views regarding preferred format of an SDM tool*		*I think you would want to access it via the Internet, I think so you can do it in our own time.* (P2, 42, one child)	*The issue with printed information is always about that it becomes outdated very quickly, and it's much harder to update.* (HP1019, Consultant)
Implementation of reproductive health shared decision‐making in routine care	Time within consultation		*No specific comments regarding time within consultation*	*I don't think the, the longer ones [SDM tools] are particularly useful to use, kind of on the day at an appointment. I don't think a patient will have a chance to kind of digest or look at really what's there during the short, kind of, clinic times that they have*. (HP1006, physiotherapist) *I think the CF specialist nurses, also they spend a lot of time with the patients as well, just getting to know more about their, their personal, like, lives and their routines, and the, and the family support network they have around them. So they really do have probably more of the stronger rapport between, between the patients, and they have a bit more time to be able to have these conversations with them.* (HP1004, physiotherapist)
	Culture that supports SDM *This sub‐theme discusses the importance of an SDM‐supportive culture and the role of SDM tools to facilitate or inhibit SDM discussions*		*I think it would be a very useful tool for younger people…looking forward to the future and starting to think, oh I need to speak… cos I didn't know I would need to speak to my CF team, I thought I could just get pregnant. I, I hadn't really considered that actually it was a huge undertaking and a huge medical undertaking and that like there was gonna be so many factors. And I think if you start with, actually there's a tool here, early on, you think, oh okay, this isn't something I can do on my own, and I think that is a good thing.* (P3, 31, 3 children)	*I think then it is about disseminating the tool, make sure we use it and we remember to use it*. (HP1023, doctor) *I think it will also empower them [patients] to have a discussion like that with their clinicians in the future. Because it will have been brought up very early on in the transplant journey. So I wouldn't underestimate the importance of a tool like that in changing culture*. (HP1023, doctor)
	Raise the topic of sexual and reproductive health early *This sub‐theme discusses when SRH conversations should be initiated*		*I mean, considering you can have children at like 16, you're probably looking at around sort of puberty, I would sort of say. I don't know, it's difficult, but maybe like 14, 15. Start talking about it*. (P6, 37, no children) *…not that there's many times when I've actually spoken to my doctor about it, but anytime that I have, it's been when I was like, 18. And that was kind of like, when the last time I was like, sort of offered to have that conversation. And like, I just felt a little bit young then, and like, I felt like, I shouldn't really be talking about that, because it felt like a bad topic. And like, that because I was young, probably it was like, I shouldn't really be thinking about having children, you know? That seems like not a very sensible idea. And I'm not sure how my CF doctor would feel about me talking about that, like, even just talking about, like contraception and like, sex and, you know, wanting to become a parent but not become a parent now. (p1, 23, no children)*	*I mean, if, forget if you have CF or don't have CF, young adults and adolescents, you know, it's important for them to be aware about family planning. And that means contraception, it means safe sex, that means pregnancy, it's everything*. (HP1019, Consultant) *I think the, from the clinician side of things, recognizing and bringing it up as something that is something that we should talk about, is something that we don't, we don't do very often, but should do. Quite early on*. (HP1017, Doctor)
	A routine sexual and reproductive health conversation *This sub‐theme encapsulates views about the value of a routine SRH conversation with a trusted HCP*.		*maybe it's part of the yearly CF review, I don't know. I mean, again, I think it's a good thing to be able to present that option and just make it aware amongst the clinic amongst, you know, men, women, or whoever, to say, look, here's this tool, you could use this, you know, if you feel like it, here's how to access it or whatnot*. (P5, 29, no children) *Yeah, we go, so every year when I have like my annual review, they normally ask then, you know, are you planning on starting a family? And they normally cover like the genetic side of it if they, you know, you want to talk about that. Yeah, so that's kind of a routine every year*. (P6, 37, no children).	*I can't imagine, sort of, saying to the patient, Oh well, I'm going to have a conversation with you about, you know, pregnancy and family planning at our appointment in a month's time, here are some tools for you to start reading through. I, I think that I, I'm much more likely to use it by me raising it at an annual review like I, I would usually do, and offering these ideally as a menu of options, offering these as tools to take away to, to think about, read and come back to us with more questions, or go away and talk to their loved ones and significant others to then have those conversations*. (HP1015, consultant)

### 3.1 Perceived Usefulness of an SDM Approach for Reproductive Health

Clinicians felt they had a good understanding of SDM. They conceptualized SDM as an opportunity to understand the patient's perspective and incorporate their values, often mentioning the importance of patient agency in the decision‐making process. Most clinicians interviewed felt their team supported SDM, but did not routinely use SDM tools. However, there was some conflating of SDM and shared care, described as the care of a patient by a multidisciplinary team, and of SDM and the use of evidence‐based practice as a basis for individualized care.

SDM‐based conversations were perceived to improve relationships between FwCF and clinicians. FwCF valued honesty from the multidisciplinary team when making health‐related decisions, and those who initiated a conversation about reproductive health found their team supportive. Clinicians described reassurance that FwCF were making an informed choice through the SDM process, suggesting that informed decision‐making through perspective‐sharing made it easier to understand and accept situations where FwCF made decisions that the multidisciplinary team might not have chosen themselves.

Providing information to support empowerment of FwCF in reproductive health decision‐making was important to both groups. However, some felt that individuals may not be open to discussing their views or be influenced by information provided, due to strongly held beliefs. FwCF described a lack of CF‐specific information for pregnancy and contraceptive choices; however, it was acknowledged that this was somewhat due to the changing landscape of CF treatment. The lack of historical CF‐specific reproductive health data was recognized by clinicians, who noted that younger FwCF not immediately planning pregnancy often asked for CF‐specific contraceptive advice. FwCF's experiences of discussions about pregnancy and reproductive health with CF teams varied, with some suggesting the subject had not been raised by either party. Information provided focused on medical aspects of pregnancy, like genetic testing and medication. FwCF with children said they would have welcomed information regarding practicalities post‐pregnancy.

### 3.2 Role of SDM Tools in Facilitating SDM

#### 3.2.1 Preparing Patients and Clinicians for a Decision‐Focussed Conversation

FwCF saw the *MyVoice:CF* tool as a resource for building knowledge before a conversation with a clinician. Some had not viewed their reproductive health as a priority in their youth, nor had they considered the implications of an accidental pregnancy. They suggested that having access to a comprehensive resource could highlight issues that they would otherwise not have considered.

Clinicians noted the value of SDM tools for demonstrating to FwCF the breadth of information needed to make an informed decision. Clinicians anticipated a benefit of improved patient knowledge of reproductive health on health outcomes, where, historically, unexpected pregnancies had caused challenges. A comprehensive tool was also seen as a useful information resource for clinicians.

### 3.3 An In‐Consultation Communication Aid

Clinicians believed that empowering FwCF to initiate conversations was beneficial, particularly where the multidisciplinary team had not proactively raised the subject or missed cues that the person wanted to discuss their reproductive health. FwCF described how being more informed increased their confidence to initiate conversations.

Using an SDM tool to guide a conversation was considered as a two‐way benefit: providing a structure for the clinician while enabling the FwCF to identify topics of relevance to them. FwCF highlighted the personal nature of reproductive health conversations, suggesting that the feature of *MyVoice:CF* enabling people to identify areas for further discussion and collating these as a list to guide a consultation, would minimize discomfort. Speaking about SDM tools in general, it was acknowledged that the structure of some tools may move the conversation in a direction that may not be appropriate for that person or raise aspects they felt uncomfortable discussing.

Participants saw SDM tools as useful in the provision of consistent information across the multidisciplinary team, with both groups sharing experiences of inconsistencies in information and in clinicians’ opinions. FwCF highlighted the benefit of a tool to collate all relevant information in one place. Clinicians echoed this, suggesting that it would reduce the risk that an important discussion point had been missed.

Tailoring information and discussing personal implications of decisions were key for both groups, with the lack of ability to personalize some tools seen as a limitation. Both liked the interactive nature of the *MyVoice:CF* tool, where the user selects the topics to explore. However, the possibility of missing important information was noted by some clinicians, as users could avoid sections they perceived as irrelevant to them. The patient‐clinician relationship was seen as key; both groups suggested that using a tool in a consultation could add further explanation or clarify misinterpretation.

### 3.4 Supporting Reflection and Deliberation

FwCF saw value in revisiting an SDM tool after a period of reflection to clarify their thoughts or refresh their understanding. Clinicians acknowledged there was often an overwhelming amount of information provided in a consultation and that reflection and revisiting the conversation were beneficial. However, they noted the danger of misinterpretation of information during asynchronous use, causing unnecessary concerns.

FwCF felt accessing the tool independently would enable them to share and discuss information with significant others, such as partners. Clinicians also recognized the importance of involving significant others.

### 3.5 Considerations for SDM Tool Development

FwCF and clinicians highlighted the importance of feeling represented and the relatability of the SDM tool content. It was suggested this could be achieved through culturally appropriate terminology and language; representations of ethnic, gender, or relationship diversity; and stories from FwCF.

Trustworthiness of both source and content was highlighted by both groups, with a trusted source disseminating the tool adding to its credibility. Clinicians noted that an online tool could be easily updated.

### 3.6 Implementation of SDM in Routine CF Care

Consultants highlighted the time constraints within appointments and the volume of information contained within comprehensive SDM tools as barriers to their use within a consultation. Nurse specialists believed they communicated more regularly with patients and had more time at each consultation.

Clinicians mentioned the importance of a culture within the team supporting SDM. One clinician highlighted the power of SDM tools to change culture, particularly to encourage patients to initiate conversations. Another cautioned against SDM tools becoming a ‘tick‐box’ exercise, closing the opportunity for authentic discussion. The importance of checking the individual's understanding and interpretation of information provided was highlighted, rather than assuming that the information has been read and understood as intended. Raising awareness of the tool amongst the multidisciplinary team was seen as an important precursor to its implementation.

FwCF and clinicians agreed that reproductive health conversations should be initiated early, with some suggesting that these should be routine conversations from the onset of puberty. The personal nature of the subject and the potential for embarrassment were highlighted as barriers, particularly for adolescents, where parents are often present in a consultation. One FwCF described a fear of being judged by clinicians for raising the subject of reproductive health at what they felt might be considered an inappropriately young age.

FwCF felt it would be useful to incorporate an SDM‐focused reproductive health‐related conversation in their annual review. For some, this was already routine practice, but the focus was typically on genetic counseling.

## 4 Discussion

SDM was seen as an integral part of routine practice by FwCF and clinicians, with both groups acknowledging the need for more regular reproductive health‐based conversations as part of CF care. SDM tools were considered valuable for both FwCF and clinicians in preparing for a conversation, guiding the conversation within a consultation, and providing a prompt for reflection and discussion with significant others.

FwCF and clinicians in this study favored starting reproductive health conversations early in puberty, with a regular conversation at annual review, supporting similar findings in the literature [25, 26]. A previous study found that while 69% of people with CF experienced SDM, it was less likely to occur with those between 18 and 24 years old [12]. Clinicians reported being supportive of empowering FwCF to initiate reproductive health‐related conversations, with FwCF suggesting that being better informed would help them feel more confident during discussions. However, others have found that while most people with CF wanted to discuss reproductive health with their CF team, 56% preferred the clinician to initiate the discussion [27] or did not feel prepared for the conversation [28]. The onus to raise the subject should not sit solely with the FwCF [12].

In their “Implement‐SDM” model, Joseph‐Williams et al identify a distinct “preparation” phase [15], also noted by LoBrutto et al in longitudinal‐SDM for chronic conditions [16]. Our findings provide further support for a preparation phase for reproductive health‐related SDM for the patient and the clinician. The importance of “trained teams” and “prepared patients” has been highlighted for SDM implementation in practice [29]. The need for trustworthy CF‐specific information was identified by FwCF and clinicians, with distribution within a multidisciplinary team and from clinicians (or a similarly trusted source) to FwCF seen as a facilitator to its use, as has been identified previously in the SDM literature as an important consideration when selecting tools for use in clinical practice [30]. As CF care is characterized by multidisciplinary input [31], there is a need for all multidisciplinary team members to provide specialism‐appropriate support for reproductive health decisions through SDM. Our study suggests that a high‐quality SDM tool, alongside clinician training, could be utilized to enable clinicians to keep abreast of a rapidly evolving field and ensure that FwCF are receiving consistent and accurate information from the whole multidisciplinary team.

LoBrutto et al suggested that, in addition to the preparation and encounter (in‐visit) stages, longitudinal‐SDM has a post‐visit processing phase [16]. Our results also highlight the value of reflection and further discussion, and as in LoBrutto's work, we noted that discussion with significant others formed a key aspect of this “processing” phase. This suggests that, for reproductive health, complex multi‐staged decisions are made, and the SDM process will be revisited and considered over time, necessitating a system that supports multiple opportunities for conversation. This need for a supportive system has been previously identified as essential for SDM implementation [29].

While FwCF focused on one SDM tool, *MyVoice:CF*, a strength of this study is that the clinicians viewed three, enabling consideration of various styles and presentation of the tool. The FwCF in this study were largely positive about the online interactive format of the *MyVoice:CF* tool; however, this may be due in part to the internet being considered an important source of information about rare diseases [32] and therefore a preference for online content in this cohort. Seeking feedback from both FwCF and clinicians was a further strength, with the views expressed being mostly congruent. In addition, the researchers conducting the analysis had varied experience in research in this area. One researcher (R.P., Health Psychologist) had previous research experience with the CF community; S.B. (clinician‐researcher) was new to the area. This encouraged reflexivity and reduced potential researcher bias. However, this study has some limitations. We adopted a collaborative approach to data analysis to ensure themes constructed from the data derive meaning that is likely to have applicability beyond the study population; however, the qualitative nature of the study does not aim to generate findings that are generalizable. Our recruitment strategy was intentionally broad and enabled participation from any geographical location in the United Kingdom, but service delivery varies between geographical regions, and ethnic minority communities were underrepresented relative to the UK general population in this study. Furthermore, the need to begin conversations about reproductive health decisions during adolescence was highlighted in our findings, but the current study focused on the views of adult females with CF (aged > 18 years). Further research to understand the views of the CF community more broadly on the implementation of reproductive health‐related SDM in routine CF care would be of benefit, including all genders, young people, people from more diverse ethnic and cultural groups, and significant others (e.g., partners and parents) of people with CF who could support them with decision‐making. Furthermore, the sample size for FwCF was disproportionately small in comparison to the clinician sample and may mean that important experiences of FwCF were not adequately represented. However, due to the rarity of the condition, the potential patient sample size was inherently limited. When considering the implications of this, we assessed information power [21] across several dimensions. Our study aim was specific and focused, building on our previous work exploring the experiences of FwCF regarding conversations about their reproductive health, collecting additional focused data to explore the role and implementation of SDM tools such as *MyVoice:CF* in a UK setting. The patient participant sample demonstrated a range of characteristics relevant to the phenomenon being studied, and the interviews gave rich, detailed dialogue, with participants giving examples and unprompted elaborations. Our analysis strategy, where we treated the patient and clinician data separately before synthesizing the data, ensured that patient voices were not lost. In comparison, due to the heterogeneity in clinician roles and locations, we recruited a larger number of clinician participants to ensure sufficient information power for this group.

## 5 Conclusions

FwCF and clinicians agree that reproductive health conversations to support SDM should be started in adolescence and revisited regularly over time. Our findings highlight the value of a comprehensive SDM tool such as *MyVoice:CF* as an aid to support reproductive health conversations at the preparation, in‐consultation, and reflection and discussion (post‐conversation) phases of longitudinal‐SDM, and for preparation and in‐consultation phases for clinicians. Further research is needed to assess the feasibility and efficacy of using reproductive health‐related SDM tools and multi‐faceted SDM interventions in CF care.

## Author Contributions

**Sarah Brown:** conceptualization, formal analysis, writing – original draft. **Denitza Williams:** conceptualization, writing – review and editing. **Jamie Duckers:** conceptualization, funding acquisition, writing – review and editing. **Traci M. Kazmerski:** writing – review and editing. **Olivia M Stransky:** writing – review and editing. **Caitlin Mason:** conceptualization, investigation, formal analysis. **Alexis Bennett:** investigation, formal analysis. **Jennifer Ward:** investigation, formal analysis. **David Taylor‐Robinson:** writing – review and editing. **Oluwaseun B Esan:** writing – review and editing. **Shantini Paranjothy:** writing – review and editing. **Daniela Schlüeter:** writing – review and editing. **Rhiannon Phillips:** conceptualization, methodology, writing – original draft, supervision.

## Conflicts of Interest

The authors declare no conflicts of interest.

## Data Availability

The data that support the findings of this study are available on request from the corresponding author. The data are not publicly available due to privacy or ethical restrictions.
